# Right Atrial Thrombus in Transient in a COVID-19 Patient: Clinical Echocardiographic Features—Case Report and Literature Review

**DOI:** 10.1007/s42399-020-00568-7

**Published:** 2020-10-08

**Authors:** Cheikh A. Abool Maaly, Jassim Al Suwaidi, Mohamad Yahya Khatib, Hakam Alzaeem

**Affiliations:** 1 Department of Cardiology and Cardiovascular Surgery, Hamad Medical Corporation (HMC), P.O Box 3050, Doha, Qatar; 2 Qatar and Weill Cornell Medical College, Doha, Qatar; 3 Critical Care and Pulmonary Medicine, HMC, Doha, Qatar

**Keywords:** COVID-19, Right atrial thrombus, Pulmonary embolism, Right heart failure

## Abstract

A 54-year-old male is admitted with COVID-19 pneumonia and received prophylactic anticoagulation. On day 8, the patient rapidly deteriorated requiring urgent endotracheal intubation. Transthoracic echocardiography revealed large right atrial thrombus in transient, resulting in pulmonary embolism and severe RV failure; fibrinolytic therapy was not effective and the patient passed away.

**Electronic supplementary material:**

The online version of this article (10.1007/s42399-020-00568-7) contains supplementary material, which is available to authorized users.

## Case Presentation

A 54-year-old male presented to the emergency department with history of fever and nonproductive cough for 1-day duration; on presentation, he was afebrile, and the patient’s heart rate is 88 beats/min, respiratory rate 16/min, and blood pressure was 154/96 mmHg. The patient had normal arterial oxygen saturation (Sao_2_) 98% on room air. Physical examination demonstrated coarse crackles in the bilateral lower lung fields. Laboratory results throughout hospitalization are shown in Table 1.

**Table 1:** Lab results

Laboratory test	Reference Values	Arrival	Hospital day 4	Hospital day 5	Hospital day 7	Hospital day 8
FiO2 (%)	21		21	80	80	91
PaO2 (mmHg)	83–108		98	96	95	88
PaO2 to FiO2 ratio	> 400		466	120	118	96.7
pH	7.35–7.45		7.45	7.46	7.47	6.94
PaCO2	35–45		32	33	33	66
Troponin T (ng/ml)	(3–15)		15	12	16	28
NT-proBNP (pg/ml)	< 125		434			
C-reactive protein (mg/dL)	0–5	261.8	353.1	274.7	232.5	276.2
Ferritin (ng/ml)	30–553	907	1.328	1.653	1.746	1.909
LDH (U/L)	135–225		482	380	441	509
White blood cells (× 109/L)	(4–10)	10.4	18.7	17.1	16.7	17
AST (U/L)	0–40	31	56	41	32	2052
ALT (U/L)	0–41	22	30	25	27	634
D-dimer	0–0.49	0.25	0.4	0.49	0.79	9.14
Fibrinogen	2–4.1		6.5	6.6	8.1	7.8
APTT	25.1–36.5		43.8	36.4	33.4	30.4

The electrocardiogram on admission showed normal sinus rhythm; chest X-ray showed bilateral patchy infiltration. He was treated with oral amoxicillin/clavulanic acid (625 mg q8 h, azithromycin (500 mg daily), hydroxychloroquine (400 mg BID), paracetamol (1000 mg TID), and sub-cutaneous enoxaparin (40 mg once daily). The patient’s fluorescence polymerase chain reaction result for SARS-CoV-2 returned positive. On the second day of admission, the patient’s fever subsided and his overall symptoms improved. On admission day 5, the patient became hypoxic with oxygen saturation of < 90% requiring supplemental oxygen inhalation initially with oxygen inhalation by non-rebreather mask at the rate of 8 l/min, which was subsequently increased to 10 l/min. On day 8, the patient suddenly developed profound hypotension and had severe desaturation requiring urgent endotracheal intubation. While D-dimer remained normal until day 8, the inflammatory parameters were progressively increasing (Table 1). Urgent bedside transthoracic echocardiography (TTE) revealed large transient thrombi in the right atrium (RA) and right ventricular (RV) with McConnell’s sign and severe RV failure (see videos). Patient urgently received fibrinolytic therapy (alteplase 100 mg) without any significant improvement while preparations are underway for VA-ECMO; unfortunately the patient had cardiac arrest and subsequently passed away.

## Discussion

Since December 2019, the COVID-19 outbreak has spread worldwide. Around 14% of cases are reported to have severe symptoms and 5% of patients developed respiratory failure, shock, and multi-organ failure. A strong correlation of COVID-19 and hypercoagulopathy has been confirmed in ICU and non-ICU COVID-19 patients [1], particularly those with limited mobility and high levels of inflammatory markers [2]. Different mechanisms contributed to the pathogenesis of hypercoagulopathy in COVID-19 disease including stasis, endothelial injury, and coagulation abnormalities such as elevated D-dimer, fibrinogen, von Willebrand factor (VWF) antigen, and factor VIII activity [3]. A retrospective study showed that microvascular pulmonary thrombosis in patient with COVID-19 pneumonia was not influenced by pre-admission treatment with anti-thrombotic therapy which may indicate a complex interplay between SARS-CoV-2 and clotting system activation [4].

The first case of COVID-19 complicated by massive pulmonary embolism (PE) and RV failure was published in JACC April 2020 [5]. Later on, many cases were reported and vast majority of them were diagnosed using computerized tomography (Table 2).

**Table 2:** Reported case reports of pulmonary embolism in patients with COVID-19

*Author*	*Country*	*Age/gender*	*COVID-pneumonia*	*Prophylactic anticoagulants*	*Day of diagnosis*	*Diagnostic modality*	*Outcome*
Julien Poissy [3]	France *Case-series*	(29 to 80) *13/M* *9/W*	*Yes*	*20 Yes* *2 No*	*?*	*CT*	*?*
*Waqas Ullah* [5]	*USA*	*59*/*W*	*Yes*	*No*	*Day before discharge*	*CT*	*Alive*
*Dany Jasinowodolinski* [6]	*Brazil*	*40/M*	*Yes*	*No*	*7*	*CT*	*?*
*Gian Battista Danzi* [7]	*Italy*	*75/W*	*Yes*	*No*	*1*	*CT*	*?*
*D.C.Rotzinger* [8]	*Switzerland*	*75*	*Yes*	*Yes*	*4*	*CT*	
*Bruno Lima Moreira* [9]	*Brazil*	*52/M*	*Yes (Lupus anticoagulant positive)*	*No*	*2*	*CT*	*Alive*
*Adriana Tamburello* [10]	*Switzerland*	*50/M*	*Yes*	*Yes*	*?*	*CT*	*?*
*Horowitz, J. M* [11]	*USA*	*62/M*	*Yes*	*Yes*	*2*	*Echocardiography* *TEE*	*Died*
*Scott E Janus* [12]	*USA*	*64/M*	*Yes*	*Yes*	*5*	*Echocardiography* *TTE*	*Alive*
*Current study*	*Qatar*	*54/M*	*Yes*	*Yes*	*8*	*Echocardiography* *TTE*	*Died*

*W* woman, *M* man

*TTE* transthoracic echocardiography

*TEE* transesophageal echocardiography

To the best of our knowledge, our case is one the first few cases of COVID-19 complicated by pulmonary embolism diagnosed by echocardiography [11, 12]. Transthoracic echocardiography is more convenient as it can be performed bedside immediately without the need to move the patient. Moreover, as far as we know, this case is one of the few cases that has developed fatal thromboembolism despite being on anticoagulants [13] and having normal D-dimer level on presentation until the day of event; this might suggest the need of higher doses of prophylactic anticoagulants in patients with severe COVID-19 pneumonia even with normal D-dimer.

## Conclusions

Fatal thrombosis may develop in patients with severe COVID-19 pneumonia despite prophylactic anticoagulation; therefore, more evidence is needed for identification of these high-risk patients and the proper doses of prophylactic anticoagulants.

### Learning Objectives

• To stress on the potential role of transthoracic echocardiography as a quick and widely available tool to help in the diagnosis of pulmonary embolism in COVID-19 patients

• To question the proper dosage of prophylactic anticoagulation in COVID-19 patients

## Electronic Supplementary Material

Video 1 Bedside Transthoracic Echocardiography. Modified Parasternal short-axis view of the heart showing a large right atrial thrombus. (MP4 77 kb)

Video 2 Bedside Transthoracic Echocardiography. Apical four chamber view of the heart showing large transient thrombi in right atrium and right ventricular with McConnell’s sign and severe RV failure. (MP4 72 kb)

Video 3 Bedside Transthoracic Echocardiography. Apical four chamber view of the heart showing large transient thrombi in right atrium and right ventricular with McConnell’s sign and severe RV failure. Doppler echocardiographic view of severe tricuspid regurgitation. (MP4 94 kb)
